# Effect of intra-coronary administration of tirofiban through aspiration catheter on patients over 60 years with ST-segment elevation myocardial infarction undergoing percutaneous coronary intervention

**DOI:** 10.1097/MD.0000000000010850

**Published:** 2018-05-25

**Authors:** Sigan Hu, Hongju Wang, Jian Zhu, Miaonan Li, Hui Li, Dasheng Gao, Heng Zhang

**Affiliations:** Department of Cardiology, the First Affiliated Hospital of Bengbu Medical College, Bengbu, Anhui, People's Republic of China.

**Keywords:** angiography, myocardial infarction, percutaneous coronary intervention, tirofiban

## Abstract

The aim of this study was to compare the efficacy and safety of 2 approaches for intra-coronary administration of tirofiban (aspiration catheter versus guiding catheter) in patients over 60 years of age undergoing percutaneous coronary intervention (PCI) for ST-segment elevation myocardial infarction (STEMI). It has been suggested that the administration of tirofiban by intra-coronary injection could promote drug absorption in the diseased region and enhance the inhibition of platelet aggregation, decreasing bleeding rates, but little is known about the comparative efficiency and safety of using guiding catheter versus aspiration catheter for delivery.

Eighty-nine patients over 60 years of age with STEMI undergoing PCI were randomly divided into 2 groups according to the injection route for intracoronary administration of tirofiban [guiding catheter (n = 41) and aspiration catheter (n = 48)]. Baseline features, epicardial and myocardial perfusion, major adverse cardiac and cerebrovascular events (MACCEs), and bleeding rate were compared.

No differences in age, gender, and history of hypertension, hypercholesterolemia, diabetes, and so on were observed (*P* > .05). The patients in the aspiration catheter group generally had a higher incidence of cerebral vascular disease. Compared with those in the guiding catheter group, patients in the aspiration catheter group obtained more favorable myocardial perfusion (*P* < .05). In-hospital and at 3-month and 6-month follow-ups, the MACCE rate and frequency of bleeding events were similar between the 2 groups (*P* > .05).

Intra-coronary delivery of tirofiban through aspiration catheter led to better myocardial perfusion in STEMI patients over 60 years of age undergoing PCI compared with intra-coronary injection of tirofiban through guiding catheter. The 2 delivery routes were associated with similar rates of MACCEs and bleeding events.

## 1 Introduction

Primary percutaneous coronary intervention (PPCI) is currently considered the most effective treatment option for ST-segment elevation myocardial infarction (STEMI).^[1,2]^ PPCI is superior to pharmacological thrombolytic reperfusion therapy if applied immediately in an experienced center. This procedure is thus recommended in patients with STEMI who can undergo PCI for the infarct-related artery (IRA) within 12 hours of symptom onset and if the door-to-balloon time can be within 90 minutes.^[3]^ Epicardial thrombolysis in myocardial infarction (TIMI) 3 flow can be achieved in the IRA in >90% of patients undergoing PPCI. However, epicardial blood flow does not necessarily equate to myocardial perfusion. After the operation, angina pectoris and even heart failure, sudden death, and other serious cardiovascular adverse events are possible. At present, it is believed that this is because sufficient and effective blood flow perfusion in the myocardium is not achieved, leading to hibernation, stunning, and necrosis of the myocardium. Thus, even with TIMI 3 flow after successful PCI may, effective myocardial perfusion may not be achieved.^[4–7]^ Excessive platelet activation and aggregation play an important role in the progression of acute coronary syndrome (ACS).^[8]^ Glycoprotein (GP) IIb/IIIa antagonists can effectively block the binding of fibrinogen to platelet glycoprotein IIb/IIIa receptor and the adhesion of platelets and damaged endothelial cells. One such antagonist, tirofiban, was shown to improve myocardial perfusion by inhibiting platelet aggregation.^[9]^ According to the ESC/ACCF/AHA guidelines, the use of GP IIb/IIIa inhibitors (GPIs) is reasonable as bailout therapy in the event of angiographic evidence of a large thrombus, slow or no reflow, or other thrombotic complications.^[10,11]^ However, the incidence of hemorrhage is also increased. Elderly patients (>60 ys of age) with coronary heart disease have complex clinical risk factors, leading to the occurrence of more complications. The incidence of bleeding is higher in these patients after strengthening of anti-platelet therapy. Tirofiban is administered through both intravenous and intra-coronary artery delivery. It has been suggested that the administration of tirofiban by intra-coronary injection can promote drug absorption in the diseased region and enhance the inhibition of platelet aggregation, thereby decreasing bleeding risk.^[12]^ Intra-coronary administration of tirofiban can be achieved using either a guiding catheter or an aspiration catheter, and little is known about the comparative efficiency and safety for these 2 delivery methods. Therefore, the present study investigated the efficacy and safety of using the 2 different methods of tirofiban administration to improve myocardial perfusion and clinical outcomes in STEMI patients >60 years of age undergoing PPCI.

## 2 Methods

### 2.1 Patient population

Between January 2012 and January 2017, a total of 369 STEMI patients underwent PPCI with 12 hours of symptom onset in our hospital. Among them, patients who were over 60 years of age were consecutively enrolled in this study. STEMI was defined as chest pain of >30 minutes duration and electrocardiogram (ECG) changes with ST segment elevation of >2 mm in at least 2 precordial leads and >1 mm in the limb leads. Patients were excluded if they had contraindications for the use of GPIs (active internal bleeding, known bleeding diathesis, intracerebral mass, or aneurysm), as were patients with cardiogenic shock at admission or patients with noncardiac conditions that could interfere with compliance with the protocol or require interruption of thienopyridine treatment. Informed consent was obtained from individual patients, and the study protocol was approved by the Institutional Review Board on Human Research. Finally, 89 patients were randomly divided into 2 groups according to the method used to administer the loading dose of tirofiban: the guiding catheter group (n = 41) and the aspiration catheter group (n = 48). The patients received. An intra-coronary injection of 10 μg/kg tirofiban (Grand Pharmaceutical Group, Wuhan, China) was applied according to the thrombus burden.

All the patients received 300 mg aspirin and 300 to 600 mg Clopidogrel and 100 U/kg unfractionated heparin (UFH) in the catheterization laboratory. PPCI was performed via the radial artery approach, using standard 6F or 7F guiding catheters. In the aspiration catheter group, patients received an intra-coronary target injection of tirofiban using the Export aspiration catheter (Medtronic, Inc.). The patients continued to receive 100 mg aspirin daily, 75 mg Clopidogrel daily (for 1 year), and 20 mg Atorvastatin daily at the physician's discretion. Other drugs, such as angiotensin converting enzyme inhibitors (ACEI) and beta blockers, were prescribed according to current guidelines and the patient's condition.

### 2.2 Observational indexes

The TIMI Myocardial Perfusion Frame Count (TMPFC) was used to standardize and quantify myocardial perfusion by timing the filling and clearance of contrast in the myocardium using cine-angiographic frame-counting.^[13–15]^ The first frame of TMPFC was defined as the frame that clearly demonstrated the first appearance of myocardial blush beyond the IRA (F_1_). The last frame of TMPFC was then defined as the frame in which contrast or myocardial blush disappeared (F_2_). The TMPFC is therefore F_2_–F_1_ frame counts at a filming rate of 15 frames/sec, or (F_2_–F_1_) × 2 frame counts at the corrected filming rate of 30 frames/sec.

TIMI grades were used to assess the forward blood flow and were graded as follows: Grade 0, no blood perfusion in the IRA and occlusion of distal vessels without blood flow; grade 1, distal stenosis of the coronary artery without blood flow; grade 2, distal stenosis of the coronary artery with blood flow, but with slow complete filling; and grade 3, blood flow similar to normal coronary artery blood flow.

The patients were followed up in the hospital and 6 months after PCI for mortality, myocardial infarction, cerebrovascular events, need for urgent revascularization, bleeding, heart failure, and hematoma. Major bleeding was defined as a >5 g/dL decrease in the hemoglobin level or need for transfusion; medium bleeding was defined as a 3 to 5 g/dL decrease in the hemoglobin level; and minor bleeding was defined as a <3 g/dL decrease in the hemoglobin level.^[16]^ Hematoma was considered significant if there was a hematoma >5 cm at the access site. Major adverse cardiac and cerebrovascular events (MACCEs) included cardiovascular mortality, myocardial infarction, heart failure, cerebrovascular accident, and need for urgent revascularization.

### 2.3 Analysis of patient data

Statistical analyses were conducted using the statistical software SPSS version 16.0 for Windows (SPSS Inc., Chicago, IL). The data are presented as mean ± standard deviation (SD) for numerical variables and raw numbers and percentages (%) for categorical variables. Continuous variables were compared using the student *t* test or nonparametric Mann-Whitney *U* test whenever the data did not appear to have a normal distribution. Categorical variables were compared using the Pearson *χ*^*2*^ or the Fisher exact test, as appropriate. *P* ≤.05 were considered statistically significant.

## 3 Results

### 3.1 Clinical characteristics and coronary angiography

The 89 STEMI patients (43 males and 46 females) enrolled in this study had a mean age of 69.9 years. A total of 89 IRAs were analyzed: 47 in the left anterior descending artery (LAD) system, 27 in the right coronary artery (RCA) system, 12 in the left circumflex artery (LCX) system, and 3 in the left main coronary artery (LM) system. Table 1 shows the baseline characteristics of patients in the 2 groups. No differences in age, gender, and history of hypertension, hypercholesterolemia, diabetes, and so on were observed. The patients in the aspiration catheter group generally had a higher incidence of cerebral vascular disease. Table 2 shows the angiographic and intervention findings in the 2 groups. No differences in the IRA, multiple vessel disease (MVD), and intervention path were observed.

**Table 1:**
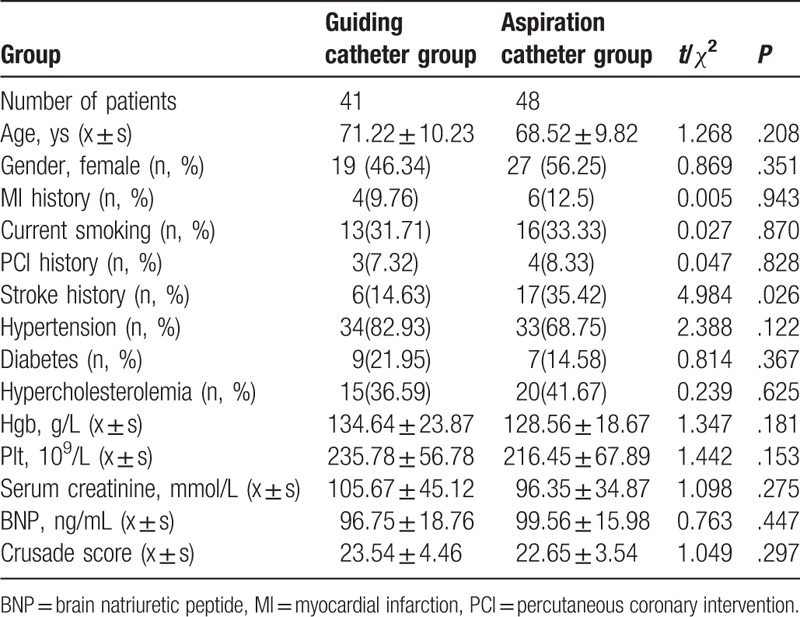
Baseline characteristics of patients.

**Table 2:**
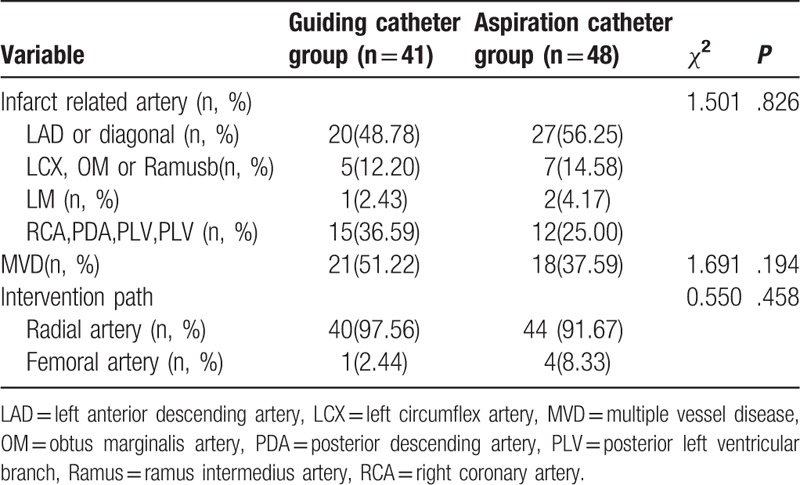
Angiographic and intervention Findings in 2 Groups.

### 3.2 Myocardial perfusion

As shown in Table 3, neither the preoperative or postoperative TIMI grades differed between the 2 groups (*P* > .05). On assessment of myocardial perfusion based on the TMPFC, patients who received intracoronary administration of tirofiban through an aspiration catheter had a lower TMPFC than those who received tirofiban through a guiding catheter (87.95 ± 12.39 vs 94.36 ± 15.87, *P* < .05).

**Table 3:**
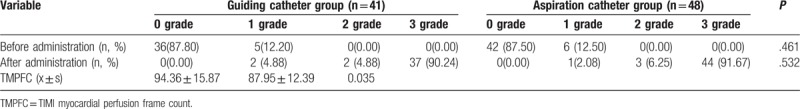
Comparison of TIMI flow grades and TMPFC between the 2 groups.

### 3.3 Prognosis and bleeding

Follow-up information was available at 6 months for all patients. The incidence of bleeding in the aspiration catheter group appeared lower than that in the guiding catheter group, but the difference was not found to be significant (*P* > .05). As shown in Table 4, no significant differences were found in the incidence of MACCEs in-hospital or at the 3-month and 6-month follow-ups.

**Table 4:**
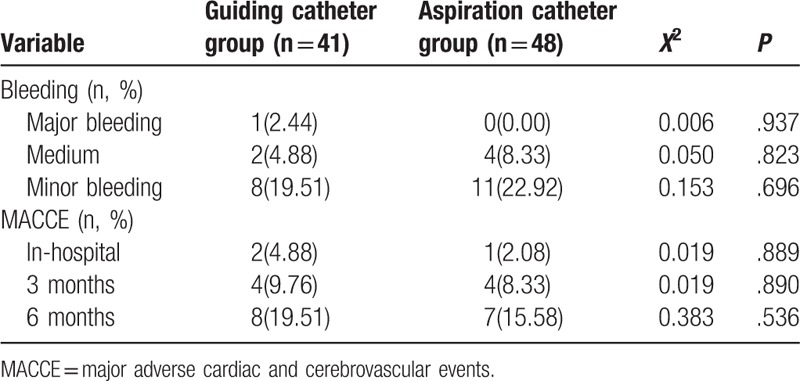
Clinical follow-up and complications in 2 groups.

## 4 Discussion

Acute myocardial infarction (AMI) is a serious type of coronary heart disease characterized by a high incidence, acute onset, and high mortality.^[17–21]^ The goal of AMI therapy is to rapidly and successfully restore epicardial blood flow and myocardial perfusion. However, full reperfusion of myocardial tissue is not achieved in some patients, even if grade TIMI 3 flow is restored in the IRA. This obviously leads to increases in the incidence of re-infarction, malignant arrhythmia, heart failure, and mortality.^[22]^ Therefore, the development of methods to improve myocardial perfusion in AMI patients is a hot topic in the field of cardiovascular research.

Compared with thrombolytic therapy, PPCI to open the IRA for STEMI patients has shown better outcomes. Slow flow or no-reflow after opening the IRA is one of the major complications of PPCI, and acute or sub-acute thrombosis is the main cause of the most serious complications and major adverse cardiac events (MACEs) after PCI.^[23]^ The incidence of slow flow in patients with AMI treated by PCI has been reported to be about 10% to 30%.^[24]^ In our study, the preoperative and postoperative TIMI grades did not differ. Therefore, the epicardial blood flow grade cannot reflect the degree of myocardial perfusion. TMPFC is a quantitative index for assessing myocardial perfusion, and it allows quantification of TIMI myocardial perfusion grading (TMPG). TMPFC was confirmed to be independent predictor of 30-day and 6-month MACCE rates. The mean TMPFC in normal arteries was shown to be 83.47 ± 17.96 frames (95% confidence interval, CI: 78.07 frames ≤TMPFC ≤88.86 frames).^[25]^ We found that patients treated with intracoronary administration of tirofiban through an aspiration catheter had a lower TMPFC than those who received tirofiban through a guiding catheter (87.95 ± 12.39 vs 94.36 ± 15.87), suggesting that administration of tirofiban through aspiration catheter would improve myocardial perfusion in STEMI patients >60 years of age undergoing PPCI, compared with intracoronary injection of tirofiban through guiding catheter. Improved myocardial perfusion was associated with improved survival of stunned myocardium, which may contribute to improved outcomes.

Tirofiban is a platelet GP IIb/IIIa inhibitor and one of the most powerful anti-platelet aggregation drugs. After administration for 5 minutes, platelet aggregation can be inhibited up to 96%, which can reduce the incidence of MACCEs. The Serbia STEMI Register study showed that tirofiban administration with PPCI, following pretreatment with 600 mg clopidogrel, improved the primary outcome after 30 days and after 1 year without an increase in major bleeding.^[26]^ Consistent with the effect of tirofiban, other GP IIb/IIIa inhibitors such as eptifibatide and abciximab improve long-term outcomes in high-risk patients with AMI following PCI. Comparisons of the need for vascular access and major bleeding complications were not possible due to low rates of these events. By modifying the route of administration of eptifibitide, the clinical effect might be preserved without increasing the risks of short-term mortality and procedural failure.^[27,28]^ A meta-analysis provided evidence of a net clinical benefit for intracoronary versus intravenous abciximab administration, with the highest benefit observed in high-risk ACS patients, such as those with reduced baseline left ventricular ejection fraction (LVEF).^[29]^

In addition, Sun et al^[30]^ found that intracoronary injection of tirofiban prevents microcirculation dysfunction during delayed PCI in AMI patients. Moreover, a meta-analysis showed that compared with intravenous administration of tirofiban, intracoronary administration of tirofiban significantly increased TIMI grade 3 flow (odds ratio [OR] = 2.11; 95% CI 1.02–4.37; *P* = .04) and TMP grade 3 flow (OR = 2.67; 95% CI 1.09–6.49; *P* = .03, *I*^*2*^ = 64%) while reducing the incidence of MACEs (OR = 0.46, 95% CI: 0.28–0.75; *P* = .002) in ACS patients.^[31]^ Intraregional administration yielded favorable outcomes in terms of myocardial tissue reperfusion as evidenced by the improved TIMI flow grade, reduced incidence of cardiac thin filament complex (CTFC), complete ST-segment resolution, and reduced incidence of MACEs without an increase in the incidence of in-hospital major bleeding events. The intralesional administration of GPIs can be recommended as the preferred regimen to guard against no-reflow.^[32]^

Our study showed that the administration of tirofiban via aspiration catheter could further improve the myocardial perfusion level in STEMI patients >60 years of age, without increasing the incidence of bleeding events. Elderly patients with coronary heart disease have complex clinical risk factors and experience more complications than younger patients. The incidence of bleeding was shown to be higher after strengthening of anti-platelet therapy, especially with intravenous administration.^[33]^ The intra-coronary administration of tirofiban can reduce the systemic effects of drugs, increasing the local drug concentration, and thus, possibly leading to better anti-platelet aggregation and anti-inflammatory effects.^[34,35]^ Intracoronary abciximab reduces the occurrence of death and MACEs in ACS.^[36]^ Moreover, the drug can be administered to the distal of end of a vascular lesion via aspiration catheter. Administration of tirofiban via an aspiration catheter can increase the drug concentration within microcirculation blood vessels to better inhibit the expression of the platelet surface receptors and cannot be cross-linked with fibrinogen.

## 5 Conclusion

Our study demonstrated that intra-coronary delivery of tirofiban through aspiration catheter may improve myocardial perfusion and long-term prognosis for STEMI patients over 60 years of age undergoing PCI compared with intracoronary injection of tirofiban through guiding catheter.

### 5.1 Study limitations

The study is limited by the small sample size; a larger study with adequate statistical power is required to validate this approach. In addition, this was a single-center clinical study, and larger prospective and multicenter studies assessing the value of this conclusion are warranted. Moreover, more precise techniques such as cardiac magnetic resonance imaging should be used to evaluate myocardial perfusion and cardiac function in future studies.

## Author contributions

**Writing – original draft:** Sigan Hu.

**Writing – review & editing:** Hongju Wang.

**Data curation:** Jian Zhu, Miaonan Li, Dasheng Gao.

**Funding acquisition:** Miaonan Li.

**Conceptualization:** Hui Li.

**Formal analysis:** Hui Li, Heng Zhang.
